# Repurposing of Furin Inhibitors to Reduce Pathogenic *E. coli*- and *Shigella flexneri*-Induced Cytotoxicity, Oxidative Stress and Inflammation in Mammalian Epithelial Cells

**DOI:** 10.3390/antibiotics14050431

**Published:** 2025-04-24

**Authors:** Isabella Rumer, Lilla Tóth, Annelie Wohlert, András Adorján, Ákos Jerzsele, Roman W. Lange, Torsten Steinmetzer, Erzsébet Gere-Pászti

**Affiliations:** 1 Department of Pharmacology and Toxicology, University of Veterinary Medicine, István utca 2, H-1078 Budapest, Hungary; 2 Department of Microbiology and Infectious Diseases, University of Veterinary Medicine, Hungária krt. 23-25, H-1143 Budapest, Hungary; 3 National Laboratory of Infectious Animal Diseases, Antimicrobial Resistance, Veterinary Public Health and Food Chain Safety, University of Veterinary Medicine, István utca 2, H-1078 Budapest, Hungary; 4 Department of Pharmacy, Institute of Pharmaceutical Chemistry, Philipps University, Marbacher Weg 6, 35032 Marburg, Germany

**Keywords:** furin inhibitors, enterohemorrhagic *E. coli*, IPEC-J2 cells, Shiga toxin, oxidative stress, inflammatory cytokines, *Shigella flexneri*

## Abstract

Background/Objectives: Enterobacteriaceae, including pathogenic *Shigella* (*S.*) *flexneri* and *Escherichia* (*E.*) *coli*, cause severe gastrointestinal infections through toxins like Shiga and Shiga-like toxins. Antibiotic use is often discouraged due to its potential to increase toxin effects or contribute to the development of resistance. The host protease furin is capable of activating several viral glycoproteins and bacterial toxins, thus enhancing pathogen infectivity. Methods: To assess the therapeutic potential of furin inhibitors, cultured epithelial cell models (IPEC-J2 and MDCK) were used. The effects of MI-1851 and MI-2415 were evaluated after short-term (2 h) and long-term (6 h) exposure to *S. flexneri*, enterohemorrhagic *E. coli* (EHEC), and enteropathogenic *E. coli* (EPEC). Cytotoxicity was determined using the CCK-8 assay, and the inflammatory response was assessed by measuring interleukin (IL)-6 and IL-8 levels. Additionally, extracellular hydrogen peroxide production was monitored in IPEC-J2 cells to evaluate the potential alterations in redox status. Results: Infections with EHEC, EPEC, and *S. flexneri* significantly reduced the viability of epithelial cells after 6 h of incubation. Furin inhibitors MI-1851 and MI-2415 decreased cytotoxicity and compensated for IL-6 and IL-8 overproduction in cells during infection with EHEC and *S. flexneri*, but not in cells exposed to EPEC. In addition, they alleviated oxidative stress, particularly during *S. flexneri* addition. Conclusions: The development of new antimicrobial drugs that act via alternative mechanisms and effectively manage life-threatening enterobacterial infections is of key importance. Targeting furin with inhibitors MI-1851 and MI-2415, thus blocking toxin activation, could prevent the development of antimicrobial resistance, reduce the need for antibiotics and enhance overall treatment outcomes.

## 1. Introduction

Enterobacteriaceae are associated with a diverse range of diseases affecting the gastrointestinal tract and other organ systems in humans and animals worldwide. The *Escherichia coli* (*E. coli*) strains isolated from human cases have been classified into distinct pathovars such as enteropathogenic *E. coli* (EPEC) and enterohaemorrhagic *E. coli* (EHEC) [1,2,3]. EPEC is characterised by its unique ability to form attaching and effacing lesions on intestinal epithelial cells, disrupting the microvilli structure and leading to diarrhoea. EHEC, especially serotype O157:H7, produces potent Shiga-like toxins (Stx1 and Stx2), which can result in life-threatening conditions such as haemorrhagic colitis and hemolytic uremic syndrome (HUS). Similarly, *Shigella* species, such as *Shigella dysenteriae* serotype 1 and *Shigella flexneri,* enhance their virulence by producing Shiga toxins, which inhibit host protein synthesis and contribute to extensive tissue damage [4,5].

Furin, a calcium-dependent serine endoprotease, activates certain proteins by specifically cleaving precursor peptides and proteins at multibasic amino acid sequences [6]. To date, furin-mediated cleavage has been identified as a critical process for the maturation of envelope glycoproteins of various evolutionarily diverse viral families, including *Herpesviridae*, *Coronaviridae*, *Flaviviridae*, *Togaviridae*, *Bornaviridae*, *Bunyaviridae*, *Filoviridae*, *Orthomyxoviridae*, *Paramyxoviridae*, *Pneumoviridae*, and *Retroviridae* [7,8]. The spread and infectivity of highly pathogenic avian influenza A viruses with a multibasic cleavage site in the surface protein hemagglutinin (especially certain H5 and H7 strains) can be propagated via proteolysis by the host protease furin, resulting in drastic effects on their pathogenicity [9,10]. In addition, furin inhibitors are capable of suppressing cleavage of spike (S) of severe acute respiratory syndrome coronavirus 2 (SARS-CoV-2) at the S1/S2 site, thus making furin an attractive target for therapeutic intervention in acute respiratory tract infection, COVID-19. The synthetic peptidomimetic furin inhibitor MI-1851 significantly suppressed viral replication in Calu-3 human airway epithelial cells, while the inhibition of endosomal cathepsins with E64d showed no effect [11].

Furthermore, certain bacterial toxin precursors also possess a furin recognition sequence (R-X-X-R) in their structure. Given its crucial role in activating several potent bacterial toxins and viral glycoproteins, furin is considered a potential target for drug development [12]. Toxin activation by the host membrane-associated serine protease, furin, occurs in endosomes/*trans*-Golgi or even on the cell surface, in contrast to cysteine protease, calpain, which can activate Stx1 in the cytoplasm. For example, in the case of Shiga toxin, furin cleaves the intact A-chain, enabling the A1 subunit to inhibit protein synthesis in host cells, ultimately leading to loss of cell viability [13,14,15,16].

Furin inhibitors have emerged as promising therapeutic agents for infectious diseases, targeting host proteases crucial for activating various bacterial toxins. These inhibitors prevent the proteolytic activation of toxins, such as *Pseudomonas aeruginosa* exotoxin A (ETA), diphtheria toxin, and the protective antigen protein of anthrax toxin [17,18,19], thereby reducing their pathogenicity and potentially mitigating disease severity [8,20]. For instance, hexa-d-arginine amide can efficiently suppress ETA- and anthrax toxin-mediated toxaemia in vitro and in rodents by inhibiting the required furin cleavage [21,22].

Antibiotic treatment is generally discouraged for these infections, particularly for EHEC, as it may increase Shiga toxin production and exacerbate the risk of HUS [23,24]. Furthermore, inappropriate antibiotic use promotes the development of multidrug-resistant (MDR) strains [25,26,27,28]. Several other potential therapeutic strategies have been developed, including glycolipid globotriaosylceramide receptor analogues and passive or active immunisation as prophylactic measures [29,30,31]. However, supportive care, including fluid and electrolyte replacement, remains the main pillar of treatment to prevent dehydration and other complications. Preventive measures, such as improved hygiene, proper food safety protocols, and public health campaigns, are essential to reduce the incidence of these infections. Targeted research initiatives and the development of innovative therapeutic strategies are critical for mitigating the burden of these pathogens on global health systems.

Unlike cancerous cells, non-tumorigenic cells closely mimic physiological glycosylation patterns and have a more regulated proliferation rate. These characteristics make the intestinal porcine epithelial cell line (IPEC-J2) cells originating from the jejunum of unsuckled piglets and the Madin-Darby canine kidney epithelial cell line (MDCK) suitable for studying cell functions, inflammatory processes, and drug responses. Based on our previous findings, treatment of IPEC-J2 cells with lipopolysaccharide (LPS) (1 μg/mL) resulted in significantly high interleukin (IL)-6, IL-8, TNF-*α*, and COX-2 gene expression levels in the IPEC-J2 cells [32]. It was also found that exposure to *E. coli* in a porcine cell model at a concentration of 10^6^ CFU/mL for 1 h significantly increased the production of IL-6 and IL-8, indicating an immune response activation with a simultaneous marked elevation in reactive oxygen species (ROS) levels. The probiotic *Enterococcus faecium* NCIMB 10415 significantly reduced IL-6 and IL-8 secretion and ROS production. This indicates that probiotics can mitigate the inflammatory and oxidative effects of *E. coli* infection [33,34]. In another investigation, pathogenic EHEC O157:H7 and non-pathogenic commensal *E. coli* were introduced into the lumens of human intestinal organoids (HIOs) via microinjection. Additionally, EHEC O157:H7 caused elevated levels of ROS and proinflammatory cytokines, such as IL-8 and IL-18, and disrupted the epithelial barrier compared to those exposed to commensal *E. coli* in HIOs. These findings highlight the damaging effects of pathogenic *E. coli* on intestinal epithelial integrity and inflammatory signalling pathways [35].

In our study, non-tumorigenic epithelial IPEC-J2 and MDCK cells were employed as model systems to evaluate the cytoprotective effects of two furin inhibitors, MI-1851 and MI-2415, at concentrations up to 50 µM. The efficacy of the inhibitors was tested under conditions of both short-term (2 h) and long-term (6 h) exposure to pathogenic bacteria, including EHEC, *S. flexneri*, and EPEC. Additionally, their anti-inflammatory and antioxidant properties were assessed by measuring changes in IL-6 and -8 levels and determining extracellular (EC) hydrogen peroxide (H_2_O_2_) production in inflamed IPEC-J2 monolayers. This study will provide insights into the putative beneficial effects of furin inhibition on infections caused by these enteropathogens.

## 2. Results

### 2.1. Cell Viability Measurements in Infected IPEC-J2 and MDCK Cells

**Figure 1:**
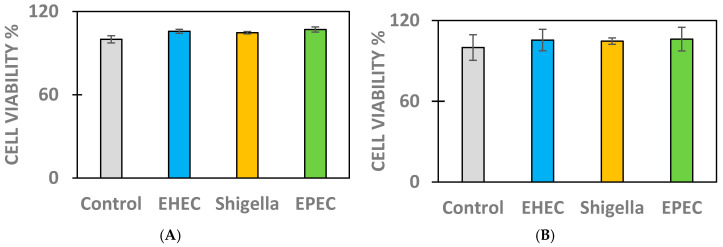
Cell viability of MDCK cells (**A**) and IPEC-J2 cells (**B**) in the absence (control) and presence of EHEC, *Shigella* spp., or EPEC. The initial bacteria concentration was 5 × 10^7^ CFU/mL, and the incubation time was 2 h in each case. The data are represented as average cell viability % ± SEM, n = 4–8 per group.

No alterations were observed in cell viability compared to controls (*p* > 0.05) in kidney and intestinal epithelial cell types after 2 h of exposure to different bacteria, such as EHEC at 5 × 10^7^ colony forming units (CFU)/mL, EPEC at 5 × 10^7^ CFU/mL, and *S. flexneri* at 5 × 10^7^ CFU/mL (Figure 1).

**Figure 2:**
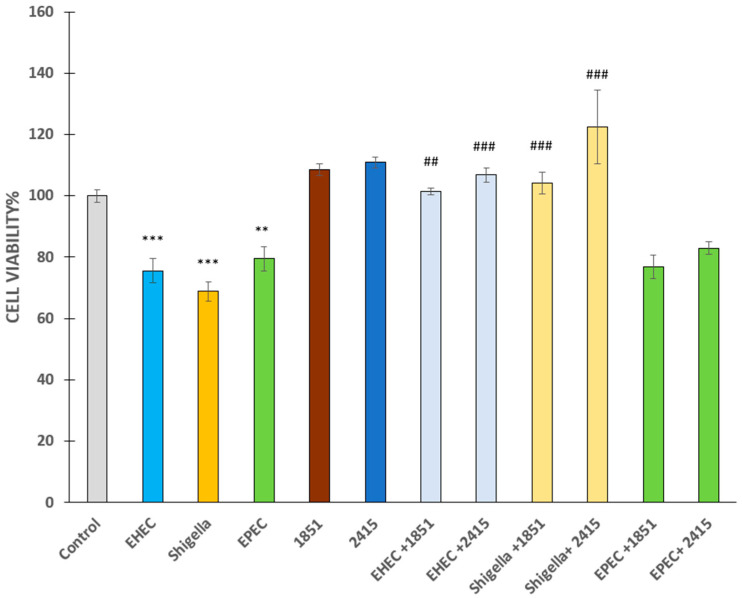
Cell viability of MDCK cells in the absence of bacteria or inhibitors (control), cells infected with the bacteria (EHEC, *Shigella* spp., or EPEC) or furin inhibitors (MI-1851 and MI-2415) alone, and after infection in the presence of furin inhibitors. Furin inhibitors were added at a concentration of 50 µM 2 h prior to and continuously during 6 h of incubation with bacteria. The initial bacteria concentration was 5 × 10^7^ CFU/mL. Lighter colours indicate the beneficial effects of the applied inhibitors. Data are represented as average cell viability ± SEM. (n = 4–8, ** *p* < 0.01, *** *p* < 0.001 compared to control and ## *p* < 0.01, ### *p* < 0.001 compared to relevant infected groups).

A 6 h infection with EHEC, *S. flexneri*, or EPEC at 5 × 10^7^ CFU/mL led to significant cell death (*p* < 0.001, *p* < 0.01, and *p* < 0.001, respectively, Figure 2). The furin inhibitors MI-1851 and MI-2415 at a concentration of 50 µM did not affect MDCK cell viability compared to the controls (*p* > 0.05). Significant protective effects of the inhibitors MI-1851 and MI-2415, which were individually added at 50 µM prior to and during infection, were observed in MDCK cells challenged with EHEC and *S. flexneri* for 6 h (*p* < 0.001, with the exception of the comparison between the EHEC and EHEC + 1851 groups, where *p* was < 0.01). In contrast, furin inhibitors (50 µM) did not alleviate the cytotoxicity caused by EPEC exposure for 6 h in MDCK cells (*p* > 0.05) (Figure 2).

**Figure 3:**
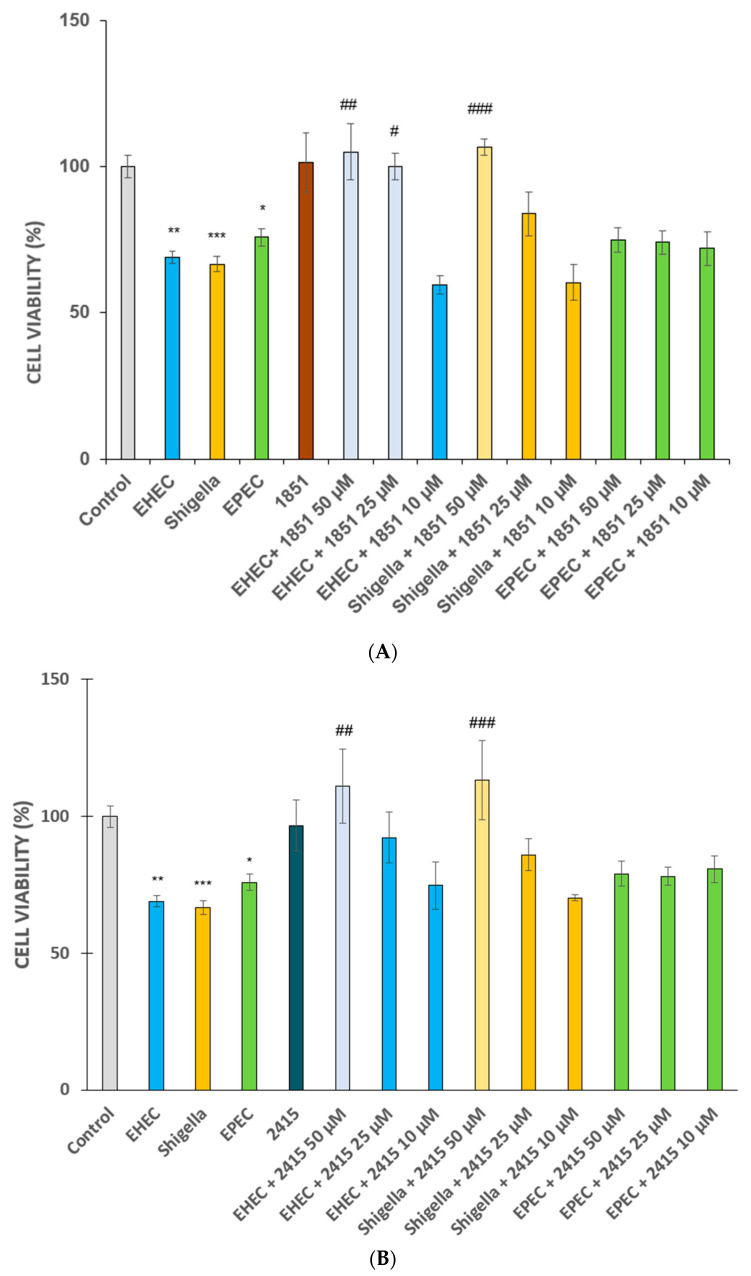
Cell viability of IPEC-J2 cells in the absence and presence of EHEC, *Shigella* spp., or EPEC with or without furin inhibitors MI-1851 (**A**) and MI-2415 (**B**). Furin inhibitors MI-1851 and 2415 were added at 50 µM for 2 h prior to and continuously during 6 h of incubation with bacteria. The starting bacteria concentration was 5 × 10^7^ CFU/mL. Lighter colours indicate beneficial effects of the applied inhibitors. The data are represented as average cell viability % ± SEM (n = 4–8, * *p* < 0.05, ** *p* < 0.01, and *** *p* < 0.001 compared to control and # *p* < 0.05, ## *p* < 0.01, ### *p* < 0.001 compared to relevant infected groups).

It was found that 6 h of infection with EHEC, EPEC, or *S. flexneri* at 5 × 10^7^ CFU/mL significantly decreased the viability of IPEC-J2 cells (*p* < 0.01, *p* = 0.0358, and *p* < 0.001, respectively). Neither furin inhibitor at 50 µM alone had exert impact on IPEC-J2 cell viability compared to controls (*p* > 0.05). Both compounds significantly protected the jejunal epithelial cells challenged with EHEC and *S. flexneri* for 6 h The inhibitor MI-1851 reduced the cytotoxicity in EHEC-treated cells in a concentration-dependent manner(at 25 µM, *p* = 0.0205 and at 50 µM, *p* < 0.01) in addition to its beneficial effects against *S. flexneri* (at 50 µM, *p* < 0.001). The inhibitor MI-2415 significantly hindered cell death only at the highest concentration of 50 µM (*p* < 0.01 comparing EHEC versus EHEC + 2415 groups and *p* <0.001 between *S. flexneri* and *S. flexneri* + 2415 groups). Similar to MDCK cells, neither furin inhibitor at 50 µM prevented cell death after EPEC infection in IPEC-J2 cells (*p* > 0.05) (Figure 3A,B).

These data confirmed that the IPEC-J2 cell line is suitable for detecting cellular changes induced by bacterial exposure and screening the putative beneficial effects of the selected furin inhibitors. Therefore, the IPEC-J2 cell line was selected for further experiments to investigate inflammatory and redox processes post-infection in the presence of furin inhibitors.

### 2.2. Changes in Proinflammatory IL-6 Secretion from IPEC-J2 Cells Post-Bacterial Infection

**Figure 4:**
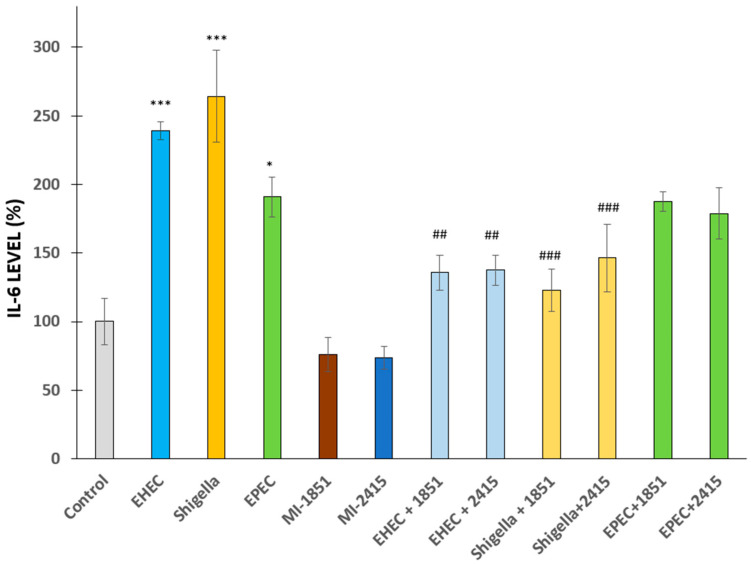
IL-6 production by IPEC-J2 cells in the absence and presence of EHEC, *Shigella* spp., or EPEC with or without furin inhibitors MI-1851 and MI-2415. Furin inhibitors were added at a concentration of 50 µM 2 h prior to and continuously during the 6 h incubation with bacteria. Lighter colours indicate beneficial effects of the applied inhibitors. The data are represented as average IL-6 % ± SEM (n = 4, * *p* < 0.05 and *** *p* < 0.001 compared to the control and ## *p* < 0.01, ### *p* < 0.01 compared to the relevant infected groups).

IL-6 is a proinflammatory cytokine that is usually excessively excreted in IPEC-J2 cells exposed to bacterial infections [33,34]. Changes in IL-6 levels were detected in IPEC-J2 cells 6 h after infection with EHEC or *S. flexneri* (*p* < 0.001 in each case) or EPEC (*p* = 0.0225) at 5 × 10^7^ CFU/mL compared to non-infected counterparts. The anti-inflammatory effects of furin inhibitors at a concentration of 50 µM, administered 2 h prior to and during bacterial exposure, were detected in bacteria-induced excessive IL-6 secretion in epithelial cells, except in the EPEC-treated groups. The inhibitor MI-1851 suppressed the increase in IL-6 levels in EHEC- (*p* < 0.01) and *S. flexneri*-mediated infections (*p* < 0.001). Similarly, the inhibitor MI-2415 was also beneficial in reducing elevated IL-6 production in cells challenged with EHEC (*p* < 0.01) or *S. flexneri* (*p* < 0.001) (Figure 4).

### 2.3. Proinflammatory IL-8 Production in IPEC-J2 Cells After Bacterial Challenge

**Figure 5:**
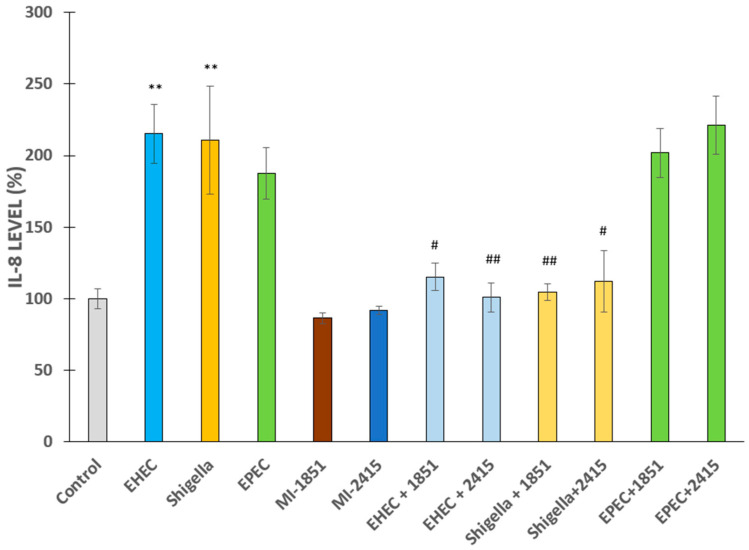
IL-8 production by IPEC-J2 cells in the absence and presence of EHEC, *Shigella* spp., or EPEC with or without furin inhibitors MI-1851 and MI-2415. The furin inhibitors were added at a concentration of 50 µM 2 h prior to and continuously during 6 h incubation with bacteria. Lighter colours indicate beneficial effects of the applied inhibitors. The data are represented as average IL-8 % ± SEM (n = 4, ** *p* < 0.01 compared to the control and # *p* < 0.05, ## *p* < 0.01 compared to the relevant infected groups).

Significant differences were observed in IL-8 levels in IPEC-J2 cells 6 h after infection with EHEC or *S. flexneri* (*p* < 0.01 in each case) but not with EPEC (*p* > 0.05) at 5 × 10^7^ CFU/mL compared to non-infected counterparts. The furin inhibitors administered at a concentration of 50 µM 2 h prior to and during bacterial exposure effectively suppressed bacteria-induced excessive IL-8 production in IPEC-J2 cells, except in the case of EPEC. EPEC infection did not induce inflammation, and furin inhibitors did not alter basal IL-8 secretion (*p* > 0.05). In contrast, the inhibitor MI-1851 alleviated the over secretion of IL-8 in EHEC (*p* = 0.0109) and *S. flexneri*-mediated infections (*p* = 0.0055). In addition, the inhibitor MI-2415 decreased the upregulated IL-8 production in cells incubated with EHEC (*p* = 0.0022) or *S. flexneri* (*p* = 0.0126) (Figure 5).

### 2.4. Extracellular Hydrogen Peroxide Release from Jejunal Epithelial Cells

**Figure 6:**
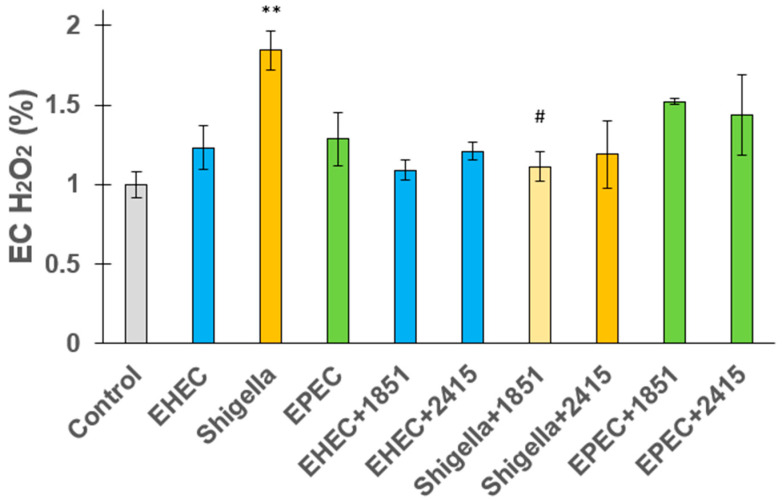
Impact of EHEC, *Shigella* spp., and EPEC on extracellular H_2_O_2_ release in IPEC-J2 cells in the presence or absence of the furin inhibitors MI-1851 and MI-2415. Hydrogen peroxide levels were measured using Amplex Red assay. *Shigella flexneri* significantly increased H_2_O_2_ production, while inhibitor MI-1851 at a concentration of 50 µM reduced this effect. The lighter orange colour indicates the protective impact of MI-1851 against *Shigella flexneri*-induced oxidative stress. Data are presented as EC H_2_O_2_% ± SEM. (n = 4, ** *p* < 0.01 compared to control and # *p* < 0.05, compared to relevant infected groups).

The release of EC H_2_O_2_ from IPEC-J2 jejunal epithelial cells was studied in response to EHEC, *S. flexneri*, and EPEC infections with and without the presence of furin inhibitors. The findings showed that bacterial infections led to higher levels of H_2_O_2_. *S. flexneri* induced the most significant oxidative response, causing a notable increase in H_2_O_2_ release (*p* = 0.00578). EHEC and EPEC also slightly increased H_2_O_2_ levels compared to the control, but the difference was not significant. EC peroxide production was significantly decreased in Shigella spp.-infected cells upon addition of the inhibitor MI-1851 at a concentration of 50 µM (*p* = 0.02505), suggesting that furin may be involved in the oxidative response triggered by this pathogen. However, furin inhibition did not significantly influence H_2_O_2_ release in cells infected with EHEC or EPEC (*p* > 0.05) (Figure 6).

## 3. Discussion

In the recent decade, antimicrobial resistance (AMR) has become an emerging risk factor that needs to be addressed in the future. The European Union (EU) One Health approach places special emphasis on the interconnectedness of human, animal, and environmental health and highlights the need for integrated strategies to address public health risks associated with zoonotic diseases, AMR, and environmental pollutants. The eradication of infections with the *Enterobacteriaceae* family, including *E. coli*, *Klebsiella* spp., and *Salmonella* spp. mainly found in livestock necessitates the administration of alternatives to antibiotic treatments due to the emergence of MDR genes [36]. The frequent occurrence of AMR poses a threat to human health [37]. Colistin, as one of the ‘last-line’ drugs against MDR Gram-negative bacteria, has been widely used for the control of neonatal and post-weaning diarrhoea in pigs; however, resistance of the Enterobacteriaceae family, mainly *E. coli*, in livestock as well as in game animals to this critically important antimicrobial drug is growing due to the presence of the mcr-1 and 2 genes [38,39,40].

In addition to propagating the effects of the proprotein convertase furin in virus spread, several studies have also found that furin plays a role in the activation of Shiga toxin, Shiga-like toxins of *E. coli*, the protective antigen (PA) of anthrax toxin, ETA from *Pseudomonas aeruginosa*, and diphtheria toxin. Therefore, furin inhibitors originally designed to prevent the cleavage of viral glycoproteins could be repurposed as antibiotics [20,41]. The efficacy of certain substrate-analogue furin inhibitors, including the 4-amidinobenzylamide (Amba)-derived inhibitor MI-1851 and the aminoisoindole (Amia)-derived analogue MI-2415, has also been proven in the maintenance of cell viability of MDCK cells upon *Pseudomonas aeruginosa* infection [42].

In addition, Shiga and Shiga-like toxins expressed by certain *Shigella* spp. and *E. coli* are activated by furin; thus, their ability to halt protein synthesis can be exacerbated by this host protease [8]. The highly potent 4-(guanidinomethyl)phenylacetyl-canavanine-Val-canavanine-4-amidinobenzylamide-type furin inhibitor showed significant antiviral effects against the highly pathogenic H7N1 influenza virus strain and prevented Shiga toxin activation in HEp-2 cells in a short-term study by reducing cell sensitivity [43,44].

The activation of Shiga, diphtheria, and anthrax toxins is primarily mediated by furin, although other cellular proteases can also cleave Shiga toxin in cells without furin activity, but less efficiently [15]. Our previously described inhibitor, 4-guanidinomethyl-phenylacetyl-Arg-Tle-Arg-4-amidinobenzylamide (MI-1148), inhibits furin with a Ki value of 5.5 pM and was tested in cell culture for its protective effect against both bacteria [41]. The efficacy against furin-dependent Shiga toxin activation in cell cultures was previously investigated using the structurally related inhibitor MI-701 (4-(guanidinomethyl) phenylacetyl-Arg-Val-Arg-4-amidinobenzylamide) [44]. It exhibited a significant dose-dependent protective effect against the cytotoxicity induced by Shiga toxin in vitro. Thus, furin appears to be a key target for inhibiting the activation of Shiga toxin, although it cannot fully prevent cell damage.

According to several reports, the cell death rates of epithelial cell lines, such as IPEC-J2 and MDCK cells, increase upon exposure to different strains of *E. coli*. In IPEC-J2 cells, a significant reduction in cell viability was observed after exposure to 5 × 10^7^ CFU of EHEC and EPEC. However, this cytotoxic effect was attenuated by pre-treatment with exopolysaccharides from *Bifidobacterium animalis* at a concentration of 5 µg for 2 h [45,46]. In addition, EHEC has been shown to disrupt epithelial barrier integrity in MDCK cells after 16–18 h of incubation with 10^7^ CFU/0.2 mL of the pathogen. EHEC can induce attaching and effacing (A/E) lesions within 3 h of infection (10^7^ CFU/mL), which also contributes to barrier dysfunction. Notably, preincubation with *Lactobacillus rhamnosus* GG (1 h, 37 °C) effectively inhibited A/E lesion formation and preserved the barrier integrity [47]. The protective effect of *Lactobacillus* species against *E. coli* infection has also been demonstrated in IPEC-J2 cells, where these probiotic bacteria effectively prevented *E. coli*’ adhesion and enhanced cell viability in the presence of citrulline [48]. In the present study, no significant changes in cell viability were observed following a 2 h incubation with EHEC or EPEC. However, after 6 h of infection, a significant decrease in cell viability was detected in both MDCK and IPEC-J2 cells, and the administration of furin inhibitors reduced this effect in EHEC and *Shigella* infections.

Several studies have examined the influence of bacterial infection on the modulation of proinflammatory cytokines, such as IL-6 and IL-8, in IPEC-J2 cells. The inflammatory response of IPEC-J2 cells exposed to *E. coli* at a concentration of 10^6^ CFU/mL was described in a study in which there was a significant increase in the secretion of IL-6 and IL-8 after 1 h of incubation, in addition to the observed oxidative stress. The study also confirmed a notable reduction in *E. coli* exposure-mediated excessive proinflammatory cytokines, as well as decreased ROS production in the presence of *Enterococcus faecium* NCIMB 10415 and *Lactobacillus rhamnosus* DSM7133 [33,34]. Increased expression of IL-8 has been detected in enterotoxigenic *E. coli* (ETEC)-infected cells compared to the control group, but EPEC infection for 4 h did not elicit a statistically significant change in IL-8 protein levels in human colon adenocarcinoma Caco-2 cells. These findings also support the protective effects of probiotic bacteria in mitigating the adverse impacts of pathogenic infections on intestinal epithelial cell cultures [49]. However, strain-specific differences were observed in the induction of cytokines, suggesting that distinct ETEC strains have varying capabilities to stimulate host immune responses [50]. In an in vivo mouse model, infection with 10^10^ CFU/mL EPEC bacteria per mouse resulted in increased intestinal permeability, epithelial damage, and compromised barrier integrity. Consistent with these findings, proinflammatory cytokines (e.g., IL-6) were significantly elevated in the colonic and ileal tissues of infected mice compared to those of the control subjects [51]. Our experimental findings are consistent with these results, providing evidence of the destructive effect of a 6 h challenge with *E. coli* and *Shigella*, and showing that these infections significantly increased IL-6 and IL-8 production in IPEC-J2 cells to a great extent. Here, we also report that both furin inhibitors, MI-1851 and MI-2415, show a substantial reduction in the overproduction of these proinflammatory cytokines induced by exposing porcine intestinal cells to EHEC and *Shigella*. This observation validates the anti-inflammatory potential of these inhibitors. However, neither furin inhibitor caused a remarkable change in the levels of these two cytokines in EPEC-infected cells.

Short-term administration of LPS from *E. coli* O111:B4 and O127:B8, each added at a concentration of 10 µg/mL, increased the production of intracellular ROS in IPEC-J2 porcine intestinal epithelial cells, indicating that these bacterial components can trigger oxidative stress; however, a lack of extracellular H_2_O_2_ production was also detected in the same study [52]. To date, it has been reported that *S. flexneri* could impact the redox balance of jejunal intestinal cells after 6 h of infection, which was efficiently alleviated by furin inhibition.

In this study, the inhibition of furin did not restore cell viability or reduce peroxide production in IPEC-J2 cells infected with EPEC. This suggests, albeit indirectly, that the modulation of furin activity does not interfere with the activation of this toxin. Despite extensive studies on EPEC virulence mechanisms, no experimental evidence is available regarding the activation of *E. coli* secreted protein C (EspC) or whether any protease-mediated cleavage events are involved to a great extent during infection. The reduced ability of EspC to induce cytopathic impacts compared to Pet toxin from enteroaggregative *E. coli* has been proven [53]. A recent study [54] investigated the crystal structure of EspC from EPEC, which plays a critical role in host cell entry and cell death. However, while the presence of a serine protease domain attached to a large β-helix with additional subdomains was confirmed in the EspC toxin, the study did not clarify any furin-like activity associated with EspC in this context.

Prevalence estimates of colistin resistance in isolates from pigs highlight the growing concern of MDR spread, while the exploration of new mechanisms of action is greatly needed to avoid the emergence of resistance. Another critically important antimicrobial, fluoroquinolone-resistant *E. coli* isolated from the enteric microbiota of patients also represents a significant healthcare challenge based on the occurrence of mutations in gyrA and parC genes, along with plasmid-mediated quinolone resistance genes [55]. Based on these findings, our aim in this study was to demonstrate that the applied furin inhibitors containing a C-terminal Amba- or Amia-residue, which have yet been applied as antimicrobial agents against *Enterobacteriaceae*, can effectively alleviate cytotoxicity, inflammation, and oxidative stress accompanied by infection with enteropathogenic bacteria in mammalian epithelial cells.

## 4. Materials and Methods

### 4.1. Bacterial Strains

EHEC (prototypic, O157:H7 SAKAI), *Shigella flexneri* and EPEC (prototypic 2348/69) strains were obtained from the bacterial collection of Dr. Domonkos Sváb at HUN-REN-VMRI (Budapest, Hungary) and preserved on beads at −80 °C. Bacteria were kept frozen at −80 °C in Microbank tubes until the beginning of the investigations, when they were propagated in Dulbecco’s modified Eagle’s medium (DMEM) for 18–24 h at 37 °C with 5% CO_2_, which are optimal conditions for culturing kidney and intestinal epithelial cells. The concentration of overnight bacterial suspensions was determined by CFU counting. The bacterial cell count used to treat the cell cultures was 5 × 10^7^ CFU/mL at the start of the experiments.

### 4.2. Culturing of Epithelial Cells

**Figure 7:**
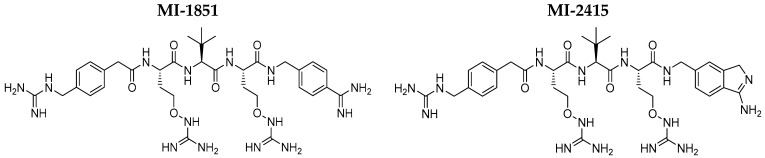
The chemical structures of the furin inhibitors used are shown. MI-1851: 4-(guanidinomethyl)-phenylacetyl-canavanine-tert. leucine—canavanine-4-amidinobenzylamide and MI-2415: 4-(guanidinomethyl)-phenylacetyl-canavanine-tert. leucine—canavanine-(amidomethyl)-1H-isoindole-3-amine.

**Figure 8:**
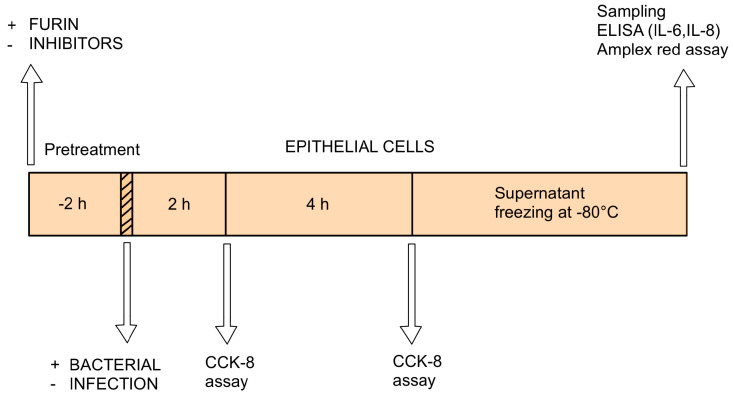
The experimental layout, including treatment types and sampling times.

The non-transformed and non-tumorigenic IPEC-J2 cell line was derived from the jejunum of neonatal unsuckled piglets. IPEC-J2 cells were obtained from Dr. Jody Gookin (Department of Clinical Sciences, College of Veterinary Medicine, North Carolina State University, Raleigh, NC, USA). The cells were cultured in DMEM and Ham’s F-12 Nutrient (1:1, DMEM/F12) with the addition of 1% insulin/selenium/transferrin, 5 ng/mL epidermal growth factor, 5% fetal bovine serum, and 1% penicillin–streptomycin. The passage numbers of the flasks ranged from 51 to 54. The cytotoxic impact of the applied bacteria and furin inhibitors was evaluated in IPEC-J2 cells seeded in 96-well plates using the CCK-8 assay (cell viability and metabolic activity). The modulating effects of the bacterial strains and furin inhibitors MI-1851 (which is identical to compound #8 in [56]) and MI-2415 (which is labelled as inhibitor #17 in [57]) on the production of proinflammatory cytokines (IL-6 and IL-8) and H_2_O_2_ release in cell supernatants were further analysed with the aid of porcine-specific ELISA (IL determination) and Amplex Red method (EC H_2_O_2_ measurement). Plain DMEM/F12 medium was used as the control. MDCK cells were maintained in DMEM supplemented with 10% fetal calf serum, antibiotics, and glutamine. Using 96-well plates, MDCK cells were seeded and grown until >95% confluence was reached, after which they were gently washed with PBS. MDCK cells were then subjected to the same treatment protocol as IPEC-J2 cells, including challenge with bacteria and/or furin inhibitors to evaluate cell viability based on the CCK-8 method. Figure 7 shows the structures of the applied furin inhibitors, and Figure 8 shows the experimental layout with indications of sampling time and treatment types with different cells.

### 4.3. Cell Viability Determination of IPEC-J2 and MDCK Cells

CCK-8 assay (Dojindo Molecular Technologies, Rockville, MD, USA) was used to determine cell viability upon 2 h or 6 h exposure of IPEC-J2 cells to EHEC, *S. flexneri* or EPEC (5 × 10^7^ CFU/mL in each treatment) in the presence or in the absence of inhibitors MI-1851 and MI-2415 in different concentrations (at 10, 25 and 50 µM). This assay was also applied to detect cell death rates in MDCK cells after 2 h and 6 h of infection with EHEC, *S. flexneri*, or EPEC (5 × 10^7^ CFU/mL in each case) with or without furin inhibitors at 50 µM. The cells were seeded into 96-well plates and incubated at 37 °C during treatment. After washing the wells three times with PBS, 100 µL of fresh phenol-red-free DMEM/F12 medium was added to each well, followed by the addition of 10 µL of CCK-8 reagent. The absorbance values were measured at 450 nm using a SpectraMax iD3 microplate (Molecular Devices, San José, CA, USA) reader after 2 h of incubation with the CCK-8 solution. The number of parallels was n = 4–8 for IPEC-J2 and MDCK-based cell cytotoxicity measurements, respectively.

### 4.4. Measurement of IL-6 and IL-8 Production in IPEC-J2 Cells

The cell-free supernatants of IPEC-J2 challenged with EHEC, *S. flexneri*, EPEC, and furin inhibitors MI-1851 and MI-2415, and their combinations, were measured after 6 h of incubation. Porcine-specific IL-6 and IL-8 sandwich ELISA kits (Merck, Darmstadt, Germany) were used to determine alterations in the levels of the selected cytokines. The supernatants were handled according to the instructions of the manufacturer and measured using a SpectraMax iD3 microplate reader at 450 nm. The number of parallel experiments was n = 4 for IL-6 and IL-8.

### 4.5. Assessment of EC H_2_O_2_ Release from IPEC-J2 Cells

The impact of EHEC, *S. flexneri*, and EPEC with or without furin inhibitors (50 µM MI-1851 and 50 µM MI-2415), either individually or in combination, on the redox balance of IPEC-J2 cells was evaluated. All compounds were prepared in phenol-red-free DMEM/F12 medium and exposed to cells in 96-well plates. The EC release of H_2_O_2_ in IPEC-J2 cells was assessed using the Amplex Red Hydrogen Peroxide Assay Kit (Invitrogen, Molecular Probes, Budapest, Hungary). Following incubation, 50 µL of cell-free supernatant was collected and mixed with an equal volume of Amplex Red working solution per well, following the manufacturer’s guidelines. Fluorescence intensity was detected using a Victor X2 2030 fluorometer (Perkin Elmer, Waltham, MA, USA), with excitation and emission wavelengths set at 530 and 590 nm, respectively. Cells maintained in the untreated medium served as the control group. Each Amplex Red assay was performed in quadruplicate for each experimental condition.

### 4.6. Statistical Evaluation of the Research Data

Statistical analysis of the data was performed using R Core Team (R version 4.0.4, The R Foundation for Statistical Computing, 2021). The normal distribution was tested using the Shapiro–Wilk test. Differences between groups were estimated using one-way analysis of variance (ANOVA) with post hoc Tukey’s test for multiple comparisons. Statistical significance was set at *p* < 0.05, *p* < 0.01, and *p* < 0.001.

## 5. Conclusions

Our findings suggest that furin inhibitors MI-1851 and MI-2415 are promising drug candidates for treating *EHEC* and *S. flexneri* infections. To our knowledge, this is the first study to demonstrate the beneficial effects of canavanine-containing furin inhibitors (MI-1851 and MI-2415) against EHEC- and *Shigella* spp.-induced infections in epithelial cell (IPEC-J2 and MDCK)- based models, suggesting their repurposing potential as antibacterial agents. Drug repositioning plays a crucial role within the One Health framework by providing innovative strategies to combat multidrug-resistant bacteria, thereby decreasing the occurrence of AMR and preserving the efficacy of authorised antibiotics across the human, animal, and environmental health sectors. Given the urgent need for alternatives to traditional antibiotics, targeting furin could contribute to the prevention of antimicrobial resistance and improvement of treatment outcomes for enterobacterial infections.

## Data Availability

All raw data supporting the results of the present study can be obtained from the corresponding author upon reasonable request.

## Abbreviations

The following abbreviations are used in this manuscript:

Amba	Amidinobenzylamide
Amia	Aminoisoindole
AMR	Antimicrobial resistance
CCK-8	Cell counting kit-8
CFU	Colony forming units
COVID-19	Coronavirus disease 2019
COX-2	Cyclooxygenase-2
DMEM	Dulbecco modified Eagles medium
*E. coli*	*Escherichia coli*
EC	Extracellular
EHEC	Enterohemorrhagic *Escherichia coli*
ELISA	Enzyme linked immunosorbent assay
EPEC	Enteropathogenic *Escherichia coli*
EspC	*E. coli* secreted protein C
ETA	Exotoxin A
ETEC	Enterotoxigenic *Escherichia coli*
EU	European Union
H_2_O_2_	Hydrogen peroxide
HIOs	Human intestinal organoids
HUS	Hemolytic uremic syndrome
IL-6/IL-8	Interleukin-6/-8
IPEC-J2	Intestinal porcine epithelial cell line
LPS	Lipopolysaccharide
MDCK	Madin-Darby kidney epithelial cell line
MDR	Multidrug-resistant
ROS	Reactive oxygen species
SARS-CoV-2	Severe acute respiratory syndrome coronavirus 2
SEM	Standard error of the mean
Stx1 and Stx2	Shiga-like toxins
TNF-α	Tumour necrosis factor—alpha
